# Cephalic Tetanus Following Auricular Metallic Trauma in an Adult: A Rare Clinical Case Presentation

**DOI:** 10.1155/crdi/2576339

**Published:** 2026-06-01

**Authors:** Sonia Taleb, Amel Ouyahia, Mounira Rais, Meriem Guechi

**Affiliations:** ^1^ Department of Infectious Diseases, University Hospital, Faculty of Medicine, Ferhat Abbas Setif University 1, Setif, Algeria, chru-strasbourg.fr; ^2^ Department of Infectious Diseases, University Hospital, Setif, Algeria, chru-strasbourg.fr

**Keywords:** cephalic tetanus, metallic projectile, otitis, trismus, vaccination

## Abstract

**Introduction:**

Cephalic tetanus of otitic origin is a well‐documented clinical entity. It is characterized by trismus associated with damage to one or more cranial nerves. Although rare, this form poses a real diagnostic challenge because of its often atypical clinical picture, which justifies rapid and appropriate treatment to limit morbidity and mortality.

**Case Presentation:**

We report the case of a 31‐year‐old patient, working as a welder and whose tetanus vaccination status was not up to date with recommended booster doses, who developed purulent otitis following auricular trauma caused by a metallic projectile, which was not treated. The course was marked by the appearance of cephalic tetanus, manifested by trismus associated with the involvement of the third cranial nerve (ptosis). Management includes the administration of antitetanus serum, a first dose of tetanus vaccine, and a prescription of metronidazole. The outcome was favorable.

**Conclusion:**

This case highlights the importance of tetanus vaccination, which remains the cornerstone of prevention and underscores the need for increased community awareness and appropriate training for health professionals. Cephalic tetanus, a rare complication of auricular trauma, requires prompt management to prevent serious sequelae and ensure favorable outcomes.

## 1. Introduction

Despite the availability of a vaccine known for its effectiveness, tetanus remains a major public health problem in many developing countries today. Approximately one million cases of tetanus occur worldwide each year, and more than 200,000 deaths are reported [1].

However, its prevalence and mortality are significantly lower in developed countries due to the widespread use of vaccination, means of prevention, and rapid and rigorous hospital care [2–4].

According to the World Health Organization (WHO), tetanus has become a rare disease in Algeria following the expansion of routine immunization programs. Recent WHO mortality estimates reported 52 tetanus‐related deaths in Algeria in 2020, corresponding to an age‐standardized death rate of 0.07 per 100,000 population. Algeria was also certified for the elimination of maternal and neonatal tetanus in 2018, reflecting substantial progress in national immunization coverage and preventive strategies [5].

Usually, the diagnosis of the generalized form of tetanus poses little difficulty for practitioners; the same is not true for localized forms, often fruste, whose early recognition is crucial.

The association between otitis and the secondary development of tetanus has been reported for several decades in the medical literature [6–8]. Indeed, otogenic tetanus requires increased vigilance and rapid management in order to avoid a progression to the generalized form which is potentially fatal. In our country, no study had focused on this clinical entity until now.

We report a case of cephalic tetanus in an adult following auricular trauma by a metallic projectile.

## 2. Case Presentation

A 31‐year‐old patient, a welder by profession, living in a rural area, whose history of tetanus vaccine booster is not up to date. He was admitted to the infectious diseases department for difficulty in opening the mouth with reduction of the right palpebral cleft. He had reported the notion of a trauma by metal projectile, resulting in a small approximately 2‐mm lesion on the right auricle. This lesion was associated with mild edema that had occurred 12 days earlier, leading to an untreated purulent otitis.

On admission, clinical examination revealed a symmetrical, irreducible, invincible, and painless trismus, occurring in an apyretic context and accompanied by the simultaneous onset of right‐sided ptosis. Trismus was neither associated with contracture of the abdomen and/or limbs nor with paroxysms. The Ear, Nose, and Throat (ENT) examination found a purulent otitis.

Moreover, the rest of the clinical examination was unremarkable. A laboratory assessment and an x‐ray of the chest were carried out, returning without any notable abnormalities.

The diagnosis of cephalic tetanus secondary to an otitic portal of entry was established clinically, given the presence of trismus, oculomotor nerve (III) involvement, and identification of the entry point.

In emergency, the management combined the administration of antitetanus serum of equine origin at a rate of 10,000 IU by intramuscularly injection without any incident, coupled with a dose of tetanus vaccination and the prescription of metronidazole at a rate of 1.5 g/day by intravenous route for 7 days.

The entry site was also treated (local antibiotic therapy). The mandatory declaration form was completed and sent to the Departmental Health and Social Affairs Directorate. The patient was discharged after 7 days of hospitalization.

**FIGURE 1:**
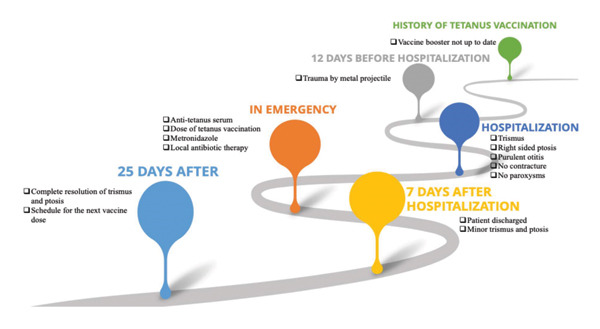
Timeline summarizing the case presentation.

The course was favorable with complete resolution of trismus and ptosis after 25 days. Tetanus vaccination was continued on an outpatient basis according to the national schedule. The clinical examination at the third month revealed no anomaly (Figure 1).

## 3. Discussion

Tetanus is a serious and potentially fatal acute infectious disease caused by the Nicolaïer’s bacillus, *Clostridium tetani*, a soil‐based, spore‐forming, and strictly anaerobic bacillus. This germ produces a powerful neurotoxin, tetanospasmin, which attaches itself to the nerve endings of motor neuron inhibitory fibers. Inhibition of these pathways leads to neuromuscular hyperexcitability that causes permanent muscle contractures [9, 10].

Globally, tetanus‐related mortality has markedly decreased over the past decades, from an estimated 200,000 annual deaths in the late 20th century to fewer than 40,000 deaths annually in recent years, mainly due to improved vaccine coverage and maternal immunization programs [11].

Cephalic tetanus is a rare and localized form of tetanus, with a short incubation period ranging from 3 to 21 days on average. In this case, it is 12 days and comfort the data of the literature [12].

It most often occurs following a wound on the head or neck. Indeed, the presence of a recent or chronic portal of entry, even minimal, as well as the lack of vaccination coverage should lead the diagnosis toward tetanus [9]. The auricular portal of entry, although rare, has been reported in the literature, particularly in connection with chronic suppurative otitis or foreign bodies [6, 13]. Soumaré et al. reported in Dakar (Senegal) a series of 55 cases of otitic tetanus, 46 of which involved patients aged between 1 and 14 years [14].

Cephalic tetanus with an otitic entry point is a form that is often underdiagnosed due to its nonspecific clinical presentation at the beginning and its relative rarity compared with generalized tetanus. Trismus dominates the clinical picture; moreover, it is the first revealing sign related to damage to the masseter muscle. Other signs may include damage to one or more cranial pairs, facial paralysis, or dysphagia [2, 15].

In this case, auricular injury caused by a metallic projectile constitutes an exceptional circumstance. Furthermore, the patient was not up to date with tetanus booster vaccination, and he was engaged in a high‐risk occupation. These factors underscore the critical importance of prevention and prompt management of craniofacial wounds, even those that appear benign, in order to prevent progression to the generalized form of the disease.

The treatment is based on several axes: neutralization of the circulating toxin by serotherapy; several serums are available: serum of equine or heterologous origin (our case) at doses of 3000 to 5000 IU in children and 10,000 IU in adults and serum of human or homologous origin which prevents serum accidents, the dose is 3000–6000 U intramuscularly [2]; antibiotic therapy (metronidazole or penicillin); management of the portal of entry if it exists; transfer to intensive care if it worsens; and especially update of the tetanus vaccination. Tetanus prevention relies on adherence to routine immunization schedules and appropriate postexposure prophylaxis following injury. Primary immunization consists of a 3‐dose series administered during infancy, followed by booster doses throughout childhood and adolescence to maintain long‐term immunity. In adults, booster vaccination is recommended at regular intervals, commonly every 10 years, to ensure sustained protection. In the setting of trauma, particularly in cases involving contaminated wounds, metallic objects, or deep tissue injury, the tetanus immunization status should be systematically assessed. Patients with complete primary vaccination with an overdue booster dose (more than 10 years have elapsed since the last vaccination) require tetanus prophylaxis according to current guidelines, including booster vaccination [16].

The clinical outcome was favorable with conventional therapy and without the need for mechanical ventilation, a circumstance that remains uncommon in the literature, where the mortality rate of the cephalic tetanus is high, sometimes exceeding 50% [17].

## 4. Conclusion

This clinical observation illustrates a typical case of cephalic tetanus in a young adult who was not up to date with the recommended booster doses of the vaccine, following a neglected entry point (post‐traumatic purulent otitis). The favorable outcome after appropriate treatment highlights the importance of rapid and multidisciplinary management, including antitetanic immunoglobulin therapy, booster vaccination, antibiotic, and treatment of the entry point.

This case also emphasizes the crucial role of prevention through vaccination and the mandatory reporting of tetanus cases. Tetanus is a preventable disease that remains severe in the absence of adequate vaccine coverage, underscoring the need for strict follow‐up through regular booster vaccination and widespread public awareness.

## Author Contributions

Sonia Taleb and Amel Ouyahia conceived the idea and conceptualized the case report.

Sonia Taleb collected the data. Sonia Taleb wrote the manuscript and revised it. Amel Ouyahia reviewed the manuscript. Mounira Rais and Meriem Guechi approved the final manuscript.

## Funding

The authors report no funding for this case report.

## Disclosure

All authors have read and approved the final manuscript.

## Ethics Statement

Approval was obtained from the Ferhat Abbas University.

## Consent

The patient gave his oral and written consent for the publication of this case report.

## Conflicts of Interest

The authors declare no conflicts of interest.

## Data Availability

The full data of this study are available from the corresponding author upon reasonable request.
